# Arteriovenous malformation in the pancreatic head initially mimicking a hypervascular mass treated with duodenum-preserving pancreatic head resection: a case report

**DOI:** 10.1186/s40792-020-01075-6

**Published:** 2020-12-01

**Authors:** Takahiro Korai, Yasutoshi Kimura, Masafumi Imamura, Minoru Nagayama, Ayumi Kanazawa, Ryo Miura, Takeshi Murakami, Daisuke Kyuno, Hiroshi Yamaguchi, Kotomi Terai, Shintaro Sugita, Takayuki Nobuoka, Tadashi Hasegawa, Ichiro Takemasa

**Affiliations:** 1 Department of Surgery, Surgical Oncology and Science, Sapporo Medical University School of Medicine, 291 Minami-1-jo Nishi 16-chome, Chuo-ku, Sapporo, Hokkaido 060-8543 Japan; 2 Surgical Pathology, Sapporo Medical University School of Medicine, 291 Minami-1-jo Nishi 16-chome, Chuo-ku, Sapporo, Hokkaido 060-8543 Japan

**Keywords:** Arteriovenous malformation, Pancreas, Duodenum-preserving pancreatic head resection

## Abstract

**Background:**

The mainstay treatment for arteriovenous malformation in the pancreatic head (Ph-AVM) is standard pancreatectomy, especially pancreaticoduodenectomy (PD), or interventional endovascular treatment. We report the first case of Ph-AVM treated with duodenum-preserving pancreatic head resection (DPPHR) performed to preserve the periampullary organs.

**Case presentation:**

A 59-year-old man presenting with back pain underwent contrast-enhanced computed tomography followed by angiography of the anterior superior pancreaticoduodenal artery. He was diagnosed with Ph-AVM and indicated for DPPHR with preservation of the periampullary organs; Ph-AVM’s benign nature seldom requires lymph node dissection. During the operation, the right colon was mobilized and the omental bursa was released to expose the periampullary structures. The pancreas was transected just above the superior mesenteric vein. The inferior pancreaticoduodenal artery and papillary arteries branching from the posterior superior pancreaticoduodenal artery were carefully preserved to maintain the blood flow to the lower bile duct and papilla of Vater. The remnant pancreas was reconstructed with pancreaticogastrostomy using the modified Blumgart method. Pathological examination of the resected specimen revealed an irregular course of the arteries and veins concomitant with marked dilation throughout the pancreatic head. The patient was pathologically diagnosed with Ph-AVM. He developed hematemesis caused by a rupture of the pseudoaneurysm on postoperative day 20 and underwent coil embolization. A bilio-enteric fistula and stenosis of the common bile duct were found and treated by placement of an endoscopic biliary stent. At the 8-month follow-up, the Ph-AVM had not recurred.

**Conclusions:**

Compared to PD, DPPHR confers the clinical benefit of preserving the periampullary organs, although further studies are needed to confirm this. Therefore, the choice of this procedure should be based on the surgical morbidities and long-term outcome of the patient.

## Background

Pancreatic arteriovenous malformation (P-AVM) is a rare disease characterized by the presence of abnormal vascular shunts from feeding arteries that drain into the portal venous system [1]. Approximately 50% of cases of P-AVM are located in the head of the pancreas [2]. The main treatments for pancreatic head AVM (Ph-AVM) are pancreaticoduodenectomy (PD) and transcatheter arterial embolization (TAE) [3]. TAE aims to control acute hemorrhage and to downgrade lesions prior to surgery, which is typically indicated for patients with poor general condition; however, recurrent bleeding occurs in 18–37% of patients after TAE owing to the development of collateral blood vessels. It is conclusively difficult to embolize multiple feeders via TAE [3, 4]. Surgical treatment is considered curative and radical, and PD is the mainstream procedure for surgical treatment of Ph-AVM [5]. There have been reports of P-AVM of the pancreatic body–tail treated with middle pancreatectomy [5] and distal pancreatectomy (DP) [6], and those of the pancreatic head with PD [5]. Duodenum-preserving pancreatic head resection (DPPHR) could be a treatment option for treating Ph-AVM, as its benign nature, unlike that of pancreatic head cancer, allows avoidance of systematic lymph node dissection. There has been no report of Ph-AVM managed with DPPHR. Here, we report the first case of Ph-AVM treated with DPPHR and review the relevant literature.

## Case presentation

**Fig. 1:**
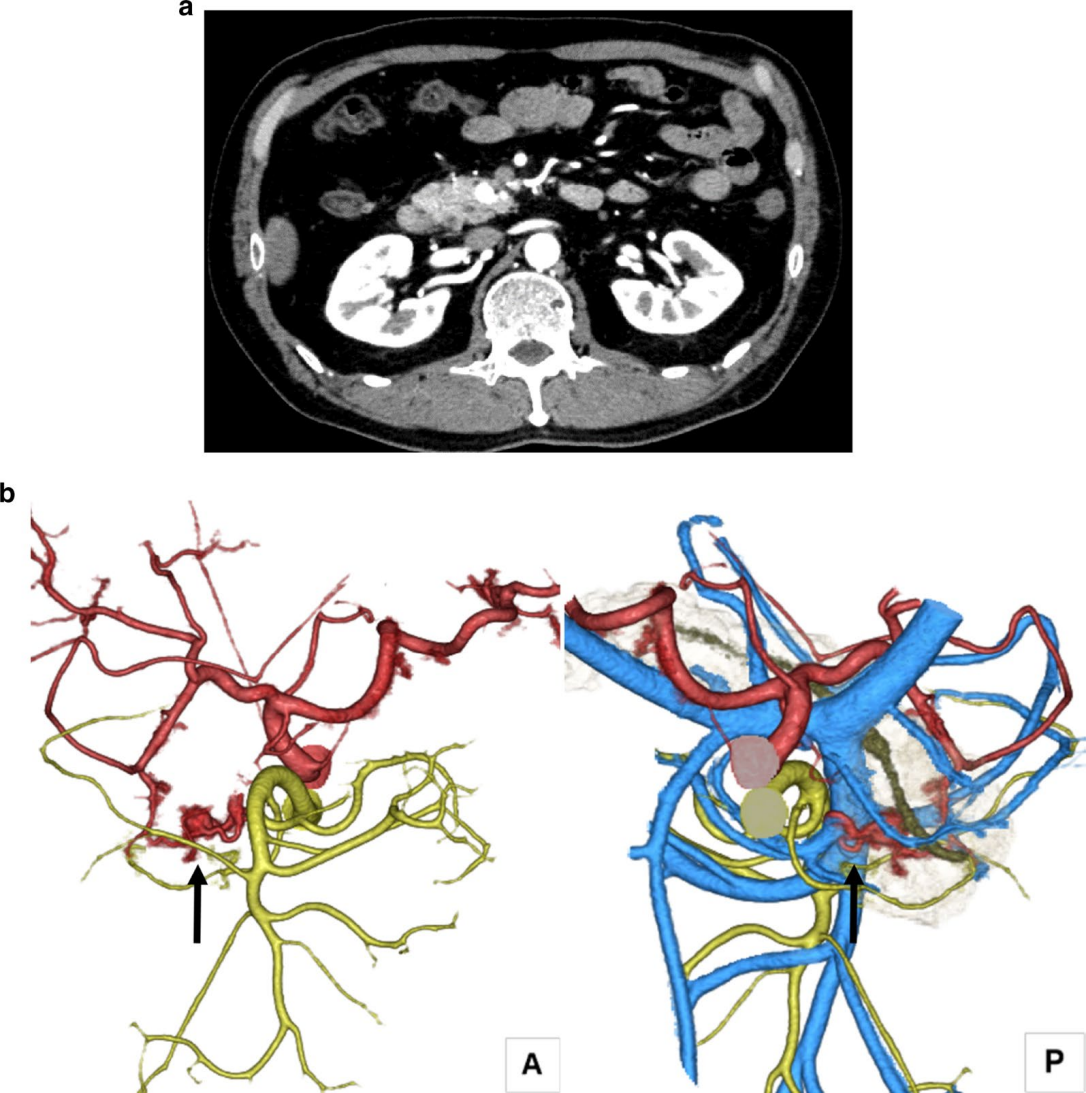
Contrast-enhanced computed tomography (CT). **a** Abdominal axial image shows a hypervascular area on the pancreatic head that stained early (arrow). **b** Arteriovenous fistula at the pancreatic head following vascular reconstruction of the contrast-enhanced CT images (arrow). *A* anterior, *P* posterior

**Fig. 2:**
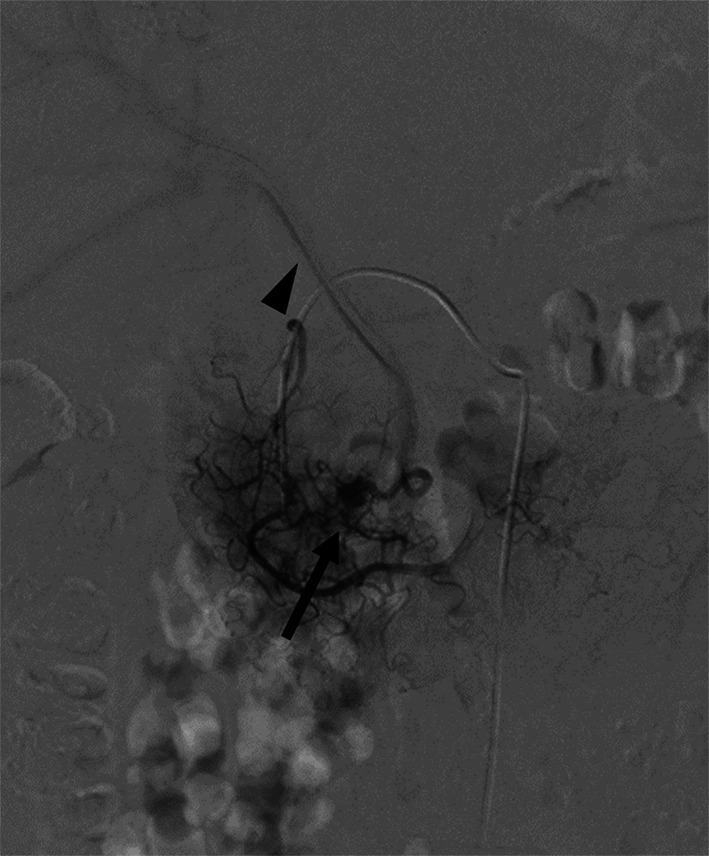
Selective angiography of the anterior superior pancreaticoduodenal artery (ASPDA). ASPDA angiography shows an abnormal vascular shunt at the pancreatic head (arrow) and early visualization of the portal vein in the early arterial phase (arrowhead)

A 59-year-old man initially presented to the referring hospital with back pain. Abdominal computed tomography (CT) revealed a pancreatic mass. The attending physician diagnosed him with a suspected neuroendocrine neoplasm, and the patient was referred to our hospital for further investigation. On physical examination, his abdomen was soft and flat, with no palpable masses. Laboratory data on admission showed that the levels of various tumor markers, including carcinoembryonic antigen and carbohydrate antigen 19-9, were within the normal ranges. Abdominal enhanced CT revealed a substantial hypervascular mass with a diameter of 9 mm that stained early on the head of the pancreas (Fig. 1a). The 3D-CT vascular reconstruction images revealed a suspected arteriovenous fistula at the pancreatic head; however, initial diagnosis was a suspected neuroendocrine neoplasm (Fig. 1b). Selective angiography of the anterior superior pancreaticoduodenal artery (ASPDA) was subsequently performed, which revealed abnormal vascular shunting in the pancreatic head along with early visualization of the portal vein at the early arterial phase (Fig. 2), indicating Ph-AVM. The nidus was confirmed by angiography and, therefore, magnetic resonance imaging was not performed. The lesion of the P-AVM was limited to the head of the pancreas, and since P-AVM is a benign disease that does not require dissection of the lymph nodes, we treated the patient using DPPHR instead of PD.

### Surgical procedures

**Fig. 3:**
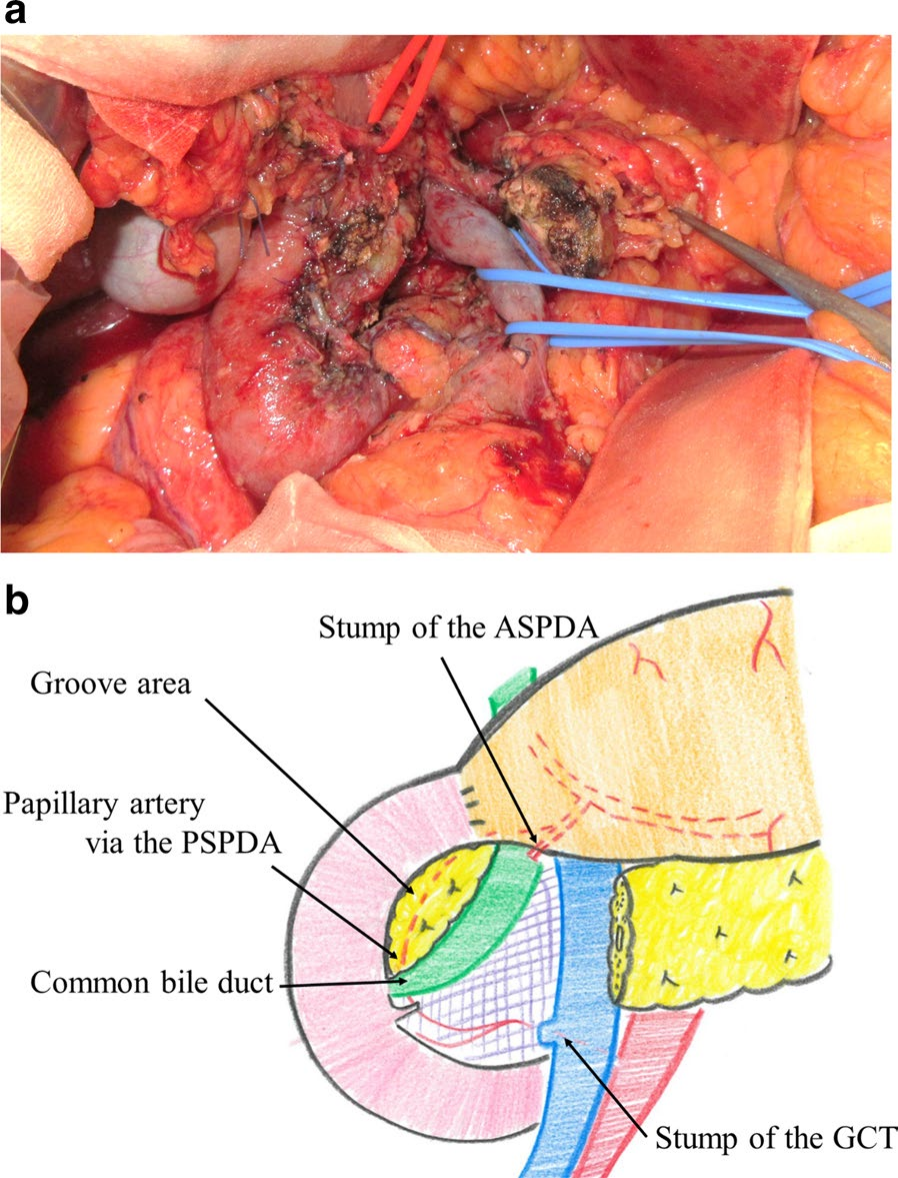
Operative findings. **a** The pancreas was dissected just above the superior mesenteric vein. The groove area and the papillary artery were preserved to maintain blood flow in the duodenum and bile duct. **b** Schema after specimen extraction and reconstruction. *ASPDA* anterior superior pancreaticoduodenal artery, *GCT* gastrocolic trunk, *PSPDA* posterior superior pancreaticoduodenal artery

A median incision was placed in the upper abdomen. The right colon was mobilized, and the omental bursa was released to expose the anterior surface of the pancreaticoduodenum. The lower part of the pancreatic uncus was released from the duodenum by detaching the pancreatic uncus using the retropancreatic fascia as an indicator. After right gastroepiploic artery branching, the pancreatic branch from the ASPDA was transected, and the gastroduodenal artery (GDA) to ASPDA was circulated and detached from the pancreas in a circumferential manner. The anterior inferior pancreaticoduodenal artery and its branches to the duodenum were carefully preserved to the lower part of the major duodenal papilla. After identification of the common duct of the major duodenal papilla, the papillary pancreatic duct and the bile duct of the papilla were encircled on the dorsal side of the pancreas, and the main pancreatic duct was ligated and dissected. The pancreas was transected just above the superior mesenteric vein with a scalpel. The groove portion of the pancreas was carefully detached from the common bile duct to preserve the posterior superior pancreaticoduodenal artery (PSPDA) and papillary artery, and then the specimen was removed (Fig. 3a, b). The remnant pancreas was anastomosed with the posterior wall of the stomach using the modified Blumgart method. The operation lasted 485 min, and the estimated blood loss was 125 ml. No intraoperative blood transfusion was required.

### Pathological findings of the resected specimen

**Fig. 4:**
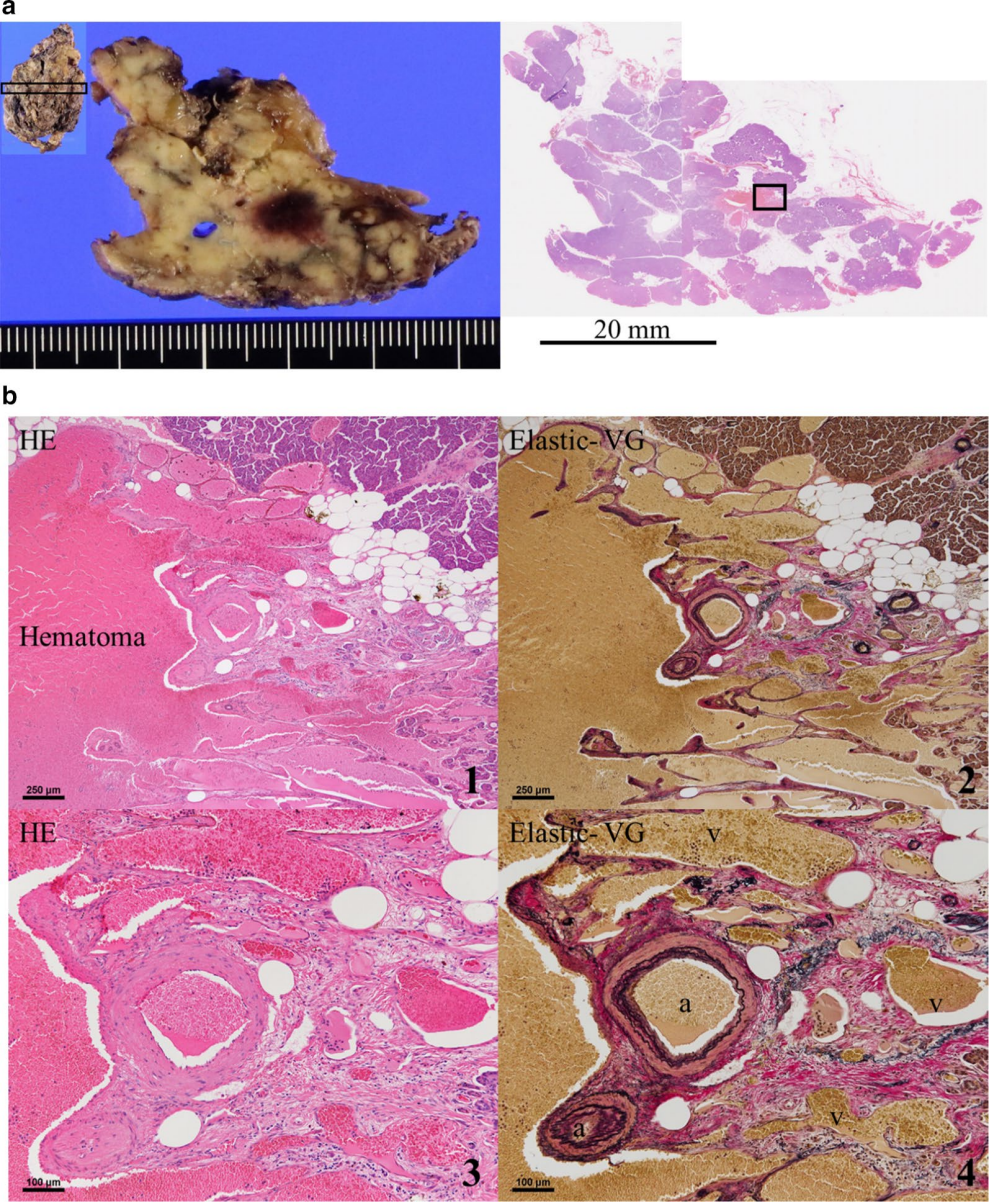
Pathological findings. **a** The whole resected specimen and partial section of the resected specimen had a dark red area, grossly 10 mm in diameter. **b** The dark red area was histologically determined to be a hematoma. Irregular running of arteries (a) and veins (v) concomitant with marked dilation were observed throughout the pancreatic head. (hematoxylin and eosin (HE), 1 × 40; 3 × 100, Elastic-van Gieson (VG), 2 × 40; 4 × 100)

The resected specimen had a dark red area, grossly 10 mm in diameter, which was histologically determined to be a hematoma. Irregular running of arteries and veins concomitant with marked dilation, as seen on the preoperative abdominal enhanced CT that revealed a substantial hypervascular mass, was observed next to the hematoma. The patient was pathologically diagnosed with P-AVM. The pancreatic ductal epithelium was not atypical, and there were no malignant findings or inflammatory changes in the specimen (Fig. 4).

### Postoperative course

**Fig. 5:**
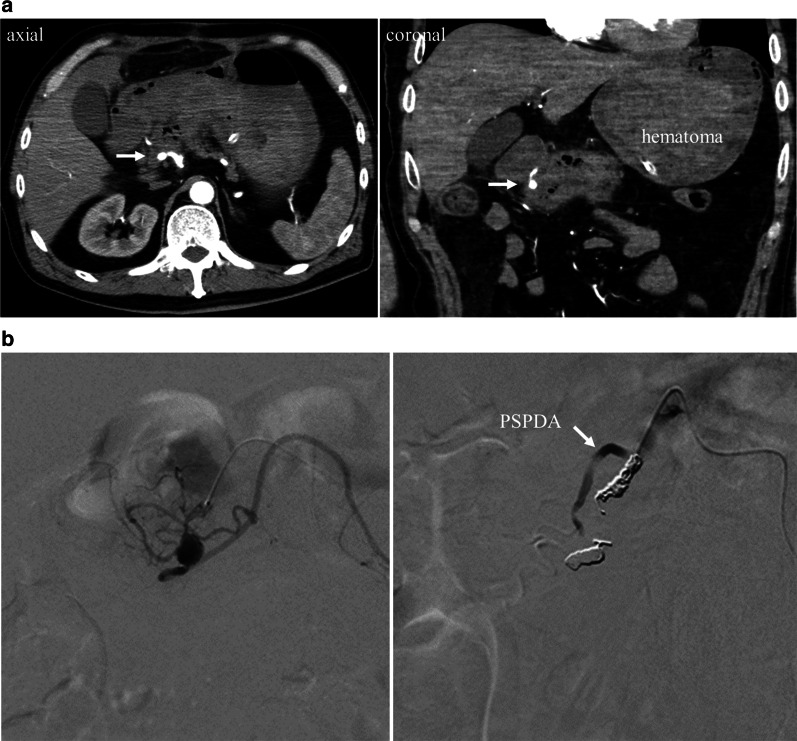
Rupture of the pseudoaneurysm after surgery. **a** Contrast-enhanced computed tomography revealed media pooled in the duodenal wall as a round 3-mm collection (arrow). **b** Angiography revealed impending rupture of the pseudoaneurysm causing gastrointestinal bleeding; therefore, the patient underwent coil embolization with preservation of the PSPDA (arrow). *PSPDA* posterior superior pancreaticoduodenal artery

**Fig. 6:**
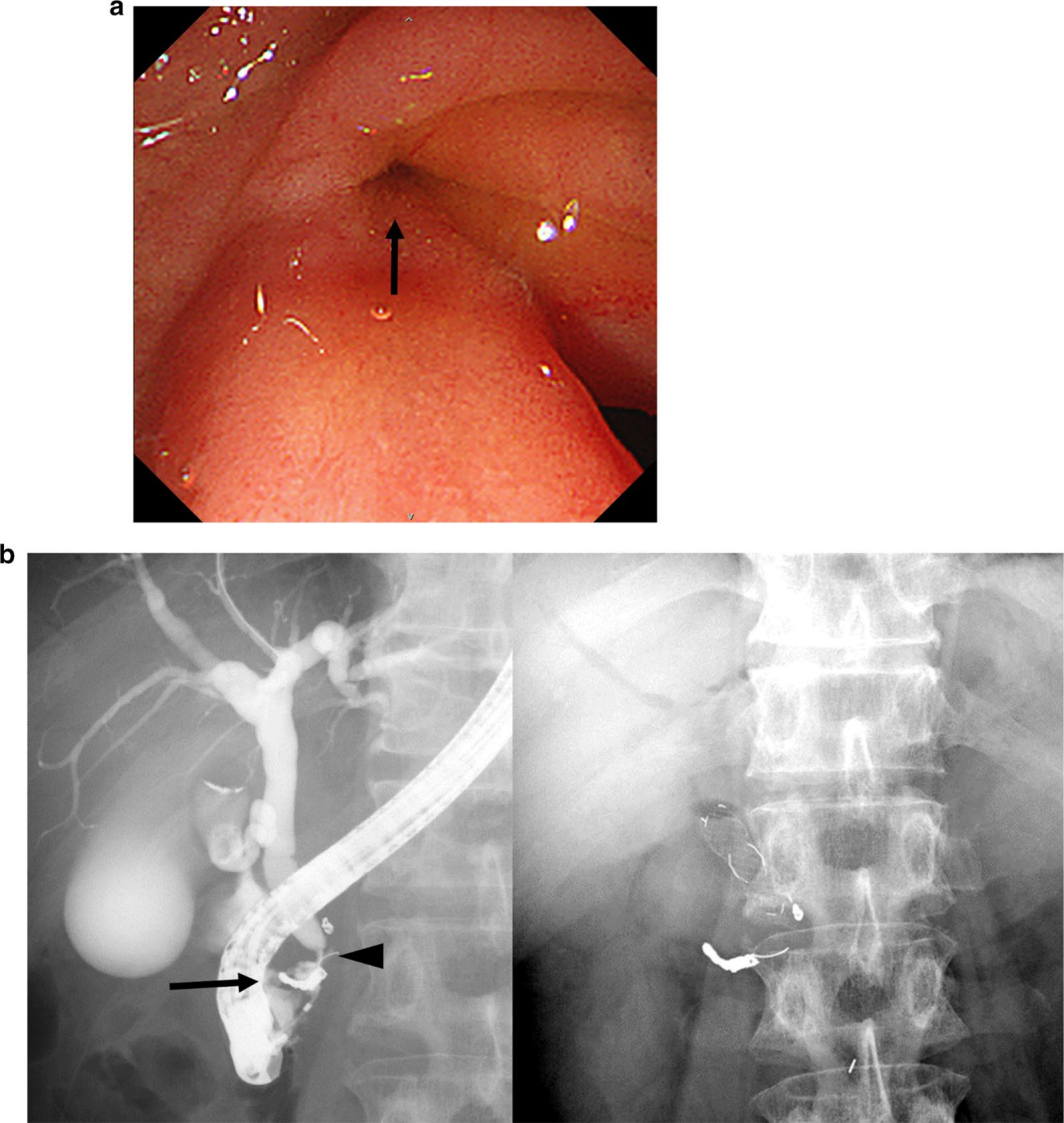
Stenosis of the common bile duct and a bilio-enteric fistula after surgery. **a** Endoscopic retrograde cholangiopancreatography (ERCP) shows a fistula with biliary excretion in the second portion of the duodenum. **b** ERCP shows stenosis of the common bile duct (arrowhead) and a bilio-enteric fistula (arrow). A bile duct stent is placed endoscopically

The patient’s postoperative course was initially uneventful, as no clinically relevant postoperative pancreatic fistula developed, and the surgical drains placed onto the pancreaticogastrostomy and para-duodenum region were subsequently removed. Based on the insulin and C-peptide level measurements, the patient’s postoperative pancreatic endocrine function was within the normal range. Although the postoperative pancreatic function test showed mildly reduced function, the patient did not present with gastrointestinal symptoms, such as fatty stool, and there was no weight loss. He received pancreatic enzyme replacement therapy to prevent the occurrence of gastrointestinal symptoms. However, he suddenly developed hematemesis classified as post-pancreatectomy hemorrhage (Grade B) [7] on postoperative day 20. Contrast-enhanced CT revealed media pooled in the duodenal wall with as a round body of 3 mm in size that connected to small vessels along with the preserved ASPDA, which was diagnosed as impending rupture of the pseudoaneurysm causing gastrointestinal bleeding; therefore, the patient underwent angiography and coil embolization, which was successfully performed at the proximal and distal ASPDA of the pseudoaneurysm (Fig. 5). He was discharged on postoperative day 30, following which, he was diagnosed with a bilio-enteric fistula located between the lower bile duct and the duodenum 4 months after the operation (Fig. 6a); endoscopic retrograde cholangiopancreatography showed stenosis of the common bile duct and a bilio-enteric fistula, which were treated by placement of an endoscopic biliary stent (Fig. 6b). At the 8-month follow-up, the patient was found to be free from any specific symptoms and the Ph-AVM had not recurred.

## Discussion

P-AVM was first reported by Halpern et al. in 1968 [8], and while it is a rare condition, the number of reported cases is increasing worldwide [2]. Congenital P-AVM accounts for 90% of all cases, resulting from abnormally differentiated remnants of primitive blood vessels [9]. Previous studies reported that 41–48% of P-AVMs are located in the pancreatic head [2, 10]. Ph-AVM causes abdominal pain, recurrent acute pancreatitis, chronic pancreatitis, jaundice, duodenal bleeding, and portal hypertension. Therefore, patients with Ph-AVM, particularly those who are symptomatic, require treatment [2, 3].

The main treatments for Ph-AVM are PD and TAE [3]. TAE is reportedly useful; however, it increases the risk of re-bleeding owing to the development of collateral blood vessels and portal hypertension [5, 11]. Therefore, TAE is useful in patients with impairment in their general condition or those who have symptoms such as gastrointestinal bleeding to control acute bleeding [2]. In our case, because the patient had localized Ph-AVM and his general condition was stable, surgery was the first-line radical treatment. In this case, the AVM was localized to the head of the pancreas, and there were no AVMs in other organs, such as the duodenum; therefore, DPPHR was selected as it is a radical surgery similar to PD.

We identified relevant articles pertaining to Ph-AVM in the English literature available on the PubMed electronic database. In total, 73 cases of Ph-AVM, including our case, have been reported on PubMed from 1968 to 2019, based on searches using “pancreas”, “head”, and “arteriovenous malformation” as keywords [4, 5, 12–22] (Table 1). Sixty-four cases involved men (87.7%) and nine involved women (12.3%). The median age of the patients was 50 years. While some reports have shown that the sizes of Ph-AVMs range from 9 mm to a maximum of 55 mm (median 13 mm), few reports actually describe the size [5]. The most common presenting symptom was epigastric pain (53.4%), followed by gastrointestinal bleeding (52.1%), back pain (6.8%), and gastrointestinal upset (5.5%). Of all cases, 4.1% were asymptomatic. The most common surgical treatment for Ph-AVM was PD (46.6%), and we found no reports of a patient with Ph-AVM having been treated with DPPHR, except for the present one. Seventeen patients who underwent TAE experienced re-bleeding or worsening portal hypertension after the treatment, resulting in 11 (64.7%) requiring surgery. We speculate that the rate of recurrence after TAE may be higher for larger Ph-AVMs; however, the size was not mentioned in most cases. The completion rate of TAE for P-AVM was found to be 38.1% [18], and the re-bleeding rate was reportedly 37% [12, 18], with a recurrence rate similar to that shown in Table 1. It is believed that Ph-AVMs with multiple inflow vessels are more likely to develop collateral vessels after TAE, leading to recurrence [12]. Of the 51 patients whose outcome was clearly stated, all survived and there were no deaths. Considering the recurrence rate after TAE, surgery is the recommended first-line radical treatment for Ph-AVM with multiple inflow vessels in patients with stable general condition.

**Table 1:** Characteristics of Ph-AVM (73 cases)

Sex	
Male	64 (87.7%)
Female	9 (12.3%)
Age (years)	
Median (range)	50 (0.6–79)
Symptom^a^	
Epigastric pain	39 (53.4%)
Gastrointestinal bleeding	38 (52.1%)
Back pain	5 (6.8%)
Gastrointestinal upset	4 (5.5%)
Body weight loss	2 (2.7%)
No symptom	3 (4.1%)
Other	7 (9.6%)
Treatment	
Surgery	
PD	34 (46.6%)
DPPHR^b^	1 (1.3%)
TAE	17 (23.3%)
Observation	17 (23.3%)
Other	4 (5.5%)
Outcome^c^	
Survival	51/51 (100%)
Recurrence	
Surgery	0/35 (0%)
TAE	11/17 (64.7%)
Death	0/51 (0%)

*Ph-AVM* pancreatic arteriovenous malformation, *PD* distal pancreatectomy, *DPPHR* duodenum-preserving pancreatic head resection, *TAE* transcatheter arterial embolization

^a^Overlapping

^b^Our case

^c^No statement for 22 cases

DPPHR, an organ-preserving pancreatectomy for chronic pancreatitis, was first reported in 1985 [23]. Unlike PD, DPPHR aims to preserve the entire duodenum and the normal biliary tree, thus reducing the incidence of recurrent postoperative cholangitis and endocrine and exocrine insufficiency. To maintain blood flow to the duodenum and bile duct during DPPHR, preserving the groove area of the pancreas sometimes contribute to reducing the risk for injuring the papillary arteries branching from the PSPDA [24]. In our case, a pseudoaneurysm ruptured at the duodenal branch of ASPDA, and coil embolization was required. Subsequently, compromised blood flow in the arcade between the inferior pancreaticoduodenal artery and GDA might have caused ischemia of the groove area at the proximal duodenum and lower bile duct. Bile duct stenosis and bilio-enteric fistula were related to this pseudoaneurysm and its embolization.

DPPHR was reported to be more effective than PD in reducing the prevalence of delayed gastric emptying, endocrine insufficiency, and the duration of postoperative hospitalization [25]. DPPHR, in this way, appears to be a favorable procedure because it eliminates digestive and absorption disorders by preserving organs and their functions with no risk of ascending cholangitis [24]. The results from a multicenter randomized controlled trial that investigated the long-term outcomes of patients who underwent DPPHR and PD for chronic pancreatitis showed no difference in postoperative complications, long-term quality of life, mortality, and morbidity between the two procedures [26]. In another report, bile duct stenosis due to residual pancreatic inflammation after DPPHR for chronic pancreatitis was found in 19.2% of patients [27], but stenosis due to bile duct ischemia has not been reported overseas. While it is difficult to compare, so far, the procedures according to the literature, it can be assumed that the incidence of postoperative complications is similar among PD and DPPHR in patient with hard pancreatic texture but slightly higher in the normal pancreatic texture. Importantly, the choice of the surgical procedure should be based on postoperative complications specific to DPPHR, such as bile duct stenosis and bilio-enteric fistula, as in this case. The clinical advantage of DPPHR, as mentioned above, remains controversial [26]. It is important to recognize that DPPHR is a technically demanding procedure and should be performed by a trained pancreatic surgeon at a well-equipped facility.

In conclusion, Ph-AVM is a rare benign condition that poses risk of gastrointestinal bleeding, pancreatitis, and portal hypertension. Surgery is recommended as a radical treatment if the patient is in good general health. In this case, Ph-AVM was successfully treated using DPPHR with organ and function preservation. The benefit of DPPHR over PD for benign pancreatic head disease is unclear, and the choice of the procedure should take into account the potential for postoperative complications.

## Data Availability

The dataset supporting the conclusions of this article is included within the article.

## Abbreviations

ASPDA: Anterior superior pancreaticoduodenal artery
CT: Computed tomography
DP: Distal pancreatectomy
DPPHR: Duodenum-preserving pancreatic head resection
GDA: Gastroduodenal artery
P-AVM: Pancreatic arteriovenous malformation
PD: Pancreaticoduodenectomy
Ph-AVM: Pancreatic head arteriovenous malformation
PSPDA: Posterior superior pancreaticoduodenal artery
TAE: Transcatheter arterial embolization
